# Efforts by the National Alliance for Caregiving

**DOI:** 10.1093/geroni/igab046.675

**Published:** 2021-12-17

**Authors:** C Grace Whiting

**Affiliations:** National Alliance for Caregiving, Washington, District of Columbia, United States

## Abstract

The National Alliance for Caregiving (NAC) conducts research, does policy analysis, develops national best-practice programs, and works to increase public awareness of family caregiving issues. In addition to national research and advocacy, NAC provides technical assistance to a national network of caregiving coalitions representing nearly 30 states. NAC recognizes that family caregivers provide important societal and financial contributions toward maintaining the well-being of those in their care. The need to support the nation’s nearly 53 million family caregivers and sustain them as the backbone of our chronic and long-term care system is a central issue in national and state efforts to reform healthcare, especially in light of the challenges presented by the COVID-19 pandemic. This presentation will provide information on the current status of these national and state efforts.

